# Psychedelics alter metaphysical beliefs

**DOI:** 10.1038/s41598-021-01209-2

**Published:** 2021-11-23

**Authors:** Christopher Timmermann, Hannes Kettner, Chris Letheby, Leor Roseman, Fernando E. Rosas, Robin L. Carhart-Harris

**Affiliations:** 1 Division of Psychiatry, Department of Brain Sciences, Centre for Psychedelic Research, Imperial College London, London, UK; 2 Department of Philosophy, The University of Western Australia, Perth, Australia; 3 Department of Philosophy, The University of Adelaide, Adelaide, Australia; 4 Data Science Institute, Imperial College London, London, UK; 5 Centre for Complexity Science, Imperial College London, London, UK; 6 Psychedelics Division, Neuroscape, Department of Neurology, University of California, San Francisco, USA

**Keywords:** Human behaviour, Social behaviour, Randomized controlled trials

## Abstract

Can the use of psychedelic drugs induce lasting changes in metaphysical beliefs? While it is popularly believed that they can, this question has never been formally tested. Here we exploited a large sample derived from prospective online surveying to determine whether and how beliefs concerning the nature of reality, consciousness, and free-will, change after psychedelic use. Results revealed significant shifts away from ‘physicalist’ or ‘materialist’ views, and towards panpsychism and fatalism, post use. With the exception of fatalism, these changes endured for at least 6 months, and were positively correlated with the extent of past psychedelic-use and improved mental-health outcomes. Path modelling suggested that the belief-shifts were moderated by impressionability at baseline and mediated by perceived emotional synchrony with others during the psychedelic experience. The observed belief-shifts post-psychedelic-use were consolidated by data from an independent controlled clinical trial. Together, these findings imply that psychedelic-use may causally influence metaphysical beliefs—shifting them away from ‘hard materialism’. We discuss whether these apparent effects are contextually independent.

## Introduction

Metaphysics is a branch of philosophy that studies themes such as the fundamental nature of reality, consciousness, and free will^1^. Research has shown that most people hold distinct metaphysical positions—even if we are not fully aware of it^2–7^. Metaphysical beliefs interface with such basic domains as health, religion, law, politics and education^8–12^, and are entwined with a society’s culture and its stability^13^.

Paradigmatic metaphysical positions can be found in physicalism (or materialism), idealism and dualism. Proponents of physicalism maintain that the nature of reality is fundamentally physical and all mental properties derive from this basic property, the position of idealism states that all physical properties derive from a fundamental reality which is mental (e.g., an irreducible, fundamental and pervasive consciousness) and dualism states that the nature of reality consists of two separate properties (i.e., the physical and mental)^1^.

Although often held implicitly, metaphysical beliefs can become explicit during or after particularly intense life experiences or transient altered states^14,15^, such as near-death experiences^16^, meditation^17^, hypnosis^18^, experiences of ‘awe’^19^, traumatic events^15,20^, and psychedelic drug-induced experiences^21–26^.

Focusing specifically on psychedelics, recent evidence has demonstrated that psychedelics can reliably and robustly induce intense, profound, and personally meaningful experiences that have been referred to as ‘mystical-type’^27^, ‘spiritual’^28^, ‘religious’^29^, ‘existential’^30^, ‘transformative^31^, ‘pivotal’^15^ or ‘peak’^32^. Some specific facets of these potentially transformative psychedelic experiences include: a perceived transcendence of the physical bounds and laws of this ‘consensus reality’^23–26^, encounters with ‘supernatural’ beings^26,29^ and an ‘ultimate reality’^29^, and the witnessing or comprehending of spatial and temporal vastness, a sense that the ‘cosmos is fundamentally conscious’^25^ and/or that all things are essentially inter-related or connected, i.e. the so-called ‘unitive experience’^33^.

From a mechanistic perspective, the unitive experience is arguably the most tangible feature of these experiences^33,34^. It is closely related to the so-called ‘overview effect’^35^, ‘universal insight’^35^, experience of ‘awe’^19,35,36^ and ‘non-dual’ states^37^. Such experiences (often reported as inducing an ‘ontological shock’^38^) appear to have a powerful capacity for mediating major shifts in perspective^19,31,39^, including shifts in metaphysical beliefs.

Psychedelics have been found to acutely increase psychological suggestibility, likely by relaxing the confidence of held beliefs^40,41^ thereby allowing for an easier transmission of others’ implicitly and explicitly held beliefs into one’s own^42^. This phenomenon may be particularly pertinent in the context of collective psychedelic experiences^43^.

Anecdotal, qualitative and retrospective reports hint that psychedelics can change metaphysical beliefs^25,26,44^, and these shifts are often explained post-hoc as having been triggered by revelations or insights^45^. However, there have been no formal, systematic, controlled and quantitative investigations of this phenomenon^46^. It has been proposed that such investigations might advance both the scientific and philosophical understanding of the psychedelic experience and its transformative effects^47^.

To address this important knowledge gap, the present study sought to examine three key questions.

1. Can psychedelics causally affect core beliefs concerning the nature of reality, consciousness and free will?
2. What is the relationship between any such belief-changes and mental health?
3. What psychological mechanisms may be involved in the putative belief-shifts?

For this purpose, we developed a prospective survey requiring respondents to answer questions pertaining to a range of metaphysical beliefs before and after attending a ceremony in which a psychedelic compound was taken. The external validity of these findings was subsequently examined via comparison with data derived from a randomized, controlled clinical trial in major depressive disorder, in which changes in beliefs were measured following psilocybin-therapy vs. a 6-week course of the selective-serotonin-reuptake-inhibitor, escitalopram.

## Results

### Non-physicalist beliefs

866 respondents completed the survey enquiring about their metaphysical beliefs at different timepoints (see “Methods”, “Supplementary Results”, and Supplementary Table 1 for sample characteristics). Thirteen items comprise the newly developed *Metaphysical Beliefs Questionnaire* and were chosen or formulated in a way to approximate classic metaphysical positions in jargon-free, non-specialist terms. The precise relationship between these items and specific philosophical positions could be contested, and so are arguably best treated at face-value. For purposes of concision and context, we did choose to ascribe some philosophical terms to the individual items and a factor dimension (Table 1) but also encourage that they be viewed as open to interpretation.

**Table 1:** Exploratory factor analysis of the Metaphysical Beliefs Questionnaire.

Item	Factor loading
**There exists another separate realm or dimension beyond this physical world that can be experienced or visited. (Ontological transcendentalism)**	**0.790**
**Visiting such immersive “realms” or “worlds” can sometimes depend on a supernatural / magical transition process or event. (Supernatural transcendentalism)**	**0.740**
**There are two separate realms of existence, the physical (body, brain and external world) and the mind, the latter being non-physical/non-material. (Dualism)**	**0.444**
There is just one primary reality: the mind and/or consciousness and all material things derive from it. (Idealism)	0.172
**There is just one primary reality: the physical; the mind (and/or consciousness) is just physical/functional properties of the brain and has an entirely material explanation. (Materialism)**	**− 0.727**
**There are other realms of existence which are more important than everyday reality. (Primacy of other realms)**	**0.592**
**The universe obeys a unifying principle which is beyond any possible material or scientific explanation. (Non-naturalism)**	**0.776**
**The universe obeys a unifying principle which is (in theory) completely addressed by a material or scientific explanation. (Naturalism)**	**− 0.598**
**The physical world is an illusion generated by consciousness or the mind. (Solipsism/Idealism)**	**0.441**
**Mind, consciousness, or soul is a fundamental quality of all things in the universe, either animate or inanimate. (Panpsychism)**	**0.642**
My conscious experience is entirely a construction of reality performed by my brain. (Internalism about consciousness)	− 0.320
My ‘self’ is entirely a construction of my brain. (Virtual self theory)	− 0.341
My experience and my ‘self’ are deeply rooted in my body and its interactions with the world and not the sole construction of my brain. (Enactivist approach to consciousness)	− 0.111

The Non-Physicalist Beliefs (NPB) factor is shown in bold.

A factor analysis on the ten self-constructed belief items developed specifically for the survey, together with three items derived from previous research^6^, revealed a single belief factor comprised of nine items, which we labelled *Non-physicalist Beliefs* (NPB; see “Methods” for factor analysis results). This single factor showed good internal consistency (Cronbach’s alpha = 0.86) and included positive loadings (> 0.4) for items related to beliefs in separate and supernatural realms of existence, a non-natural unifying principle in reality, panpsychism, dualism, and solipsism/idealism. Items that loaded negatively (< − 0.4) referred to ‘hard’ monistic materialist or physicalist positions, or a belief in natural (as opposed to super-natural) explanations for phenomena in the universe (naturalism). These items and their loadings are shown in Table 1.

**Figure 1:**
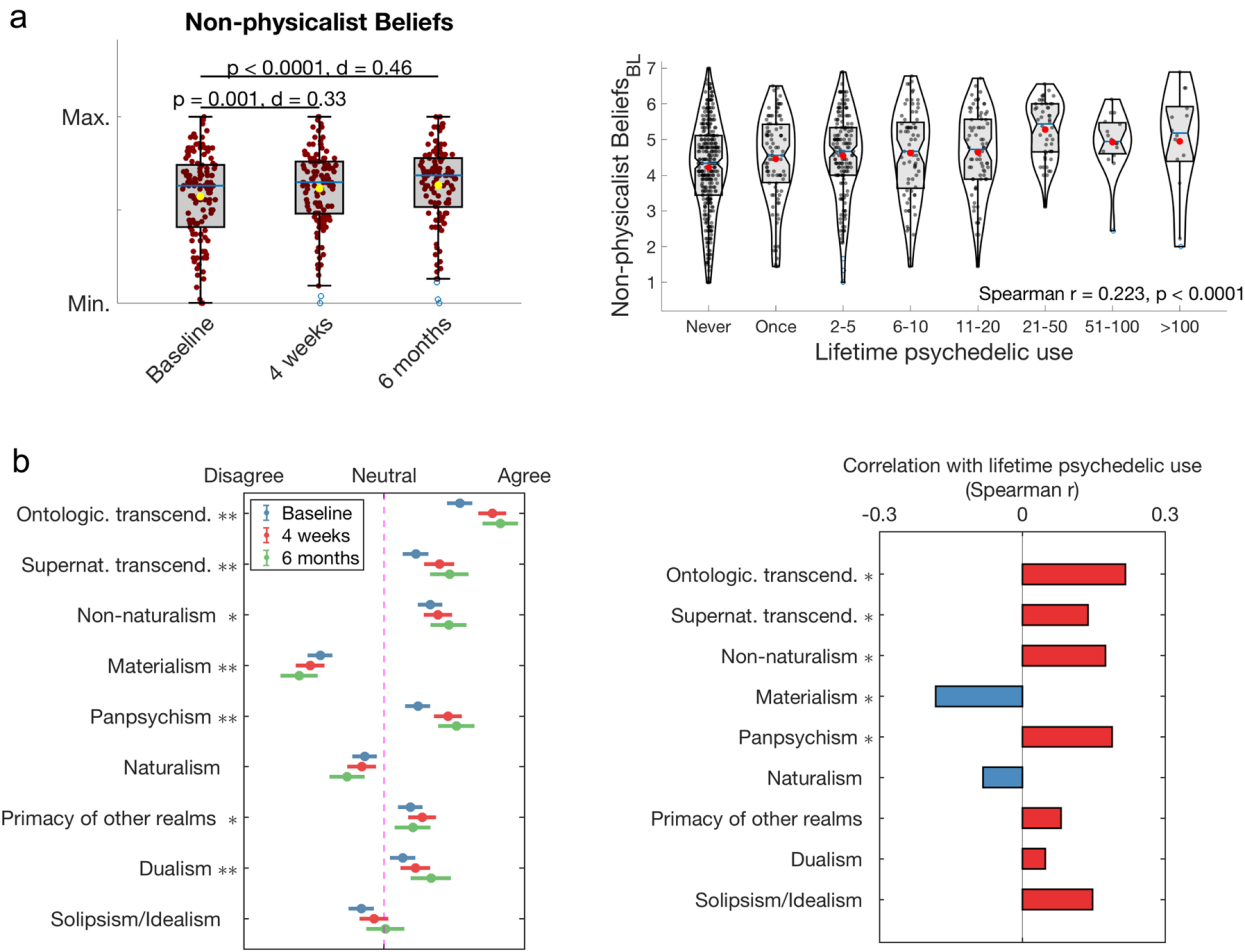
Psychedelic use is associated with shifts in metaphysical beliefs away from hard physicalism or materialism. Attending a psychedelic ceremony was associated with shifts away from hard-materialistic views (**a**-left), and items associated with transcendentalism, non-naturalism, panpsychism, primacy of other realms, dualism and solipsism/idealism (**b**-left), with some changes enduring up to 6 months (Bonferroni-corrected). Additionally, significant positive relationships were observed between lifetime psychedelic use and baseline scores on metaphysical beliefs (**a**-right), and items referring to transcendentalism, non-naturalism, and panpsychism, while a negative relationship was found with materialism (**b**-right). (**b**-left: mean values and standard errors displayed. *Significant change at 4 weeks; **significant change at 6 months, Bonferroni-corrected; **b**-right: * p < 0.0001, Bonferroni-corrected).

We compared NPB scores before attending a ceremony involving psychedelic use (baseline) with NPB scores 4 weeks and 6 months after the ceremony. Pooling scores for the NPB factor, analyses revealed a significant shift away from physicalism at 4 weeks compared with baseline (*t*(121) = 3.66, *p* = 0.001, *d* = 0.33, confidence interval, 95% CI [0.12, 0.39]). These changes were sustained 6 months after the ceremony (*t*(121) = 5.07, *p* < 0.0001, *d* = 0.46, 95% CI [0.22, 0.50]) (Fig. 1a). Larger effect sizes were found for respondents who were embarking on their first psychedelic experience (the so-called ‘psychedelic naïve’), with significant changes found at 4 weeks (*t*(52) = 3.85, *p* = 0.001, *d* = 0.53, 95% CI [0.21, 0.66]) and 6 months (*t*(52) = 5.32, *p* < 0.0001, *d* = 0.73, 95% CI [0.36, 0.80]) (Supplementary Fig. 1a). Analyses of each individual item for the NPB factor revealed increases in notions of transcendentalism, mind–body dualism, and panpsychism—among others, with some changes remaining significant for 6 months (see Fig. 1b-left and Supplementary Fig. 1b for findings for ‘naïve’ respondents). Additionally, a significant positive correlation was found between previous psychedelic use and shifts away from the hard-materialism pole of the hard-materialism vs. hard-dualism spectrum (Fig. 1b-right) at baseline (*r* = 0.223, *p* < 0.0001).

### Fatalism

Analysis of the prospective data (i.e., pre versus post ceremony) revealed that the psychedelic ceremony was associated with increases in scores of *Fatalistic determinism*^4^ (see “Supplementary Methods” for the items used) at 4 weeks versus baseline (*t*(121) = 2.81, *p* = 0.012, *d* = 0.25, 95% CI [0.06, 0.37]); however, this effect did not persist at 6 months. For psychedelic-naïve participants, larger effect sizes were detected at 4 weeks compared with baseline (*t*(52) = 3.38, *p* = 0.003, *d* = 0.46, 95% CI [0.16, 0.63]), and the changes persisted for at least 6 months (*t*(52) = 2.86, *p* = 0.012, *d* = 0.39, 95% CI [0.11, 0.64]) (Supplementary Fig. 2a). Consistent with the results described above, correlational analysis revealed a mild (*r* = 0.186) but significant positive correlation (*p* < 0.0001) between baseline beliefs in *Fatalistic Determinism* and lifetime psychedelic-use (see Supplementary Figs. 2b and 3 for correlations between scales at different timepoints).

### Conversion of preferred metaphysical beliefs

**Figure 2:**
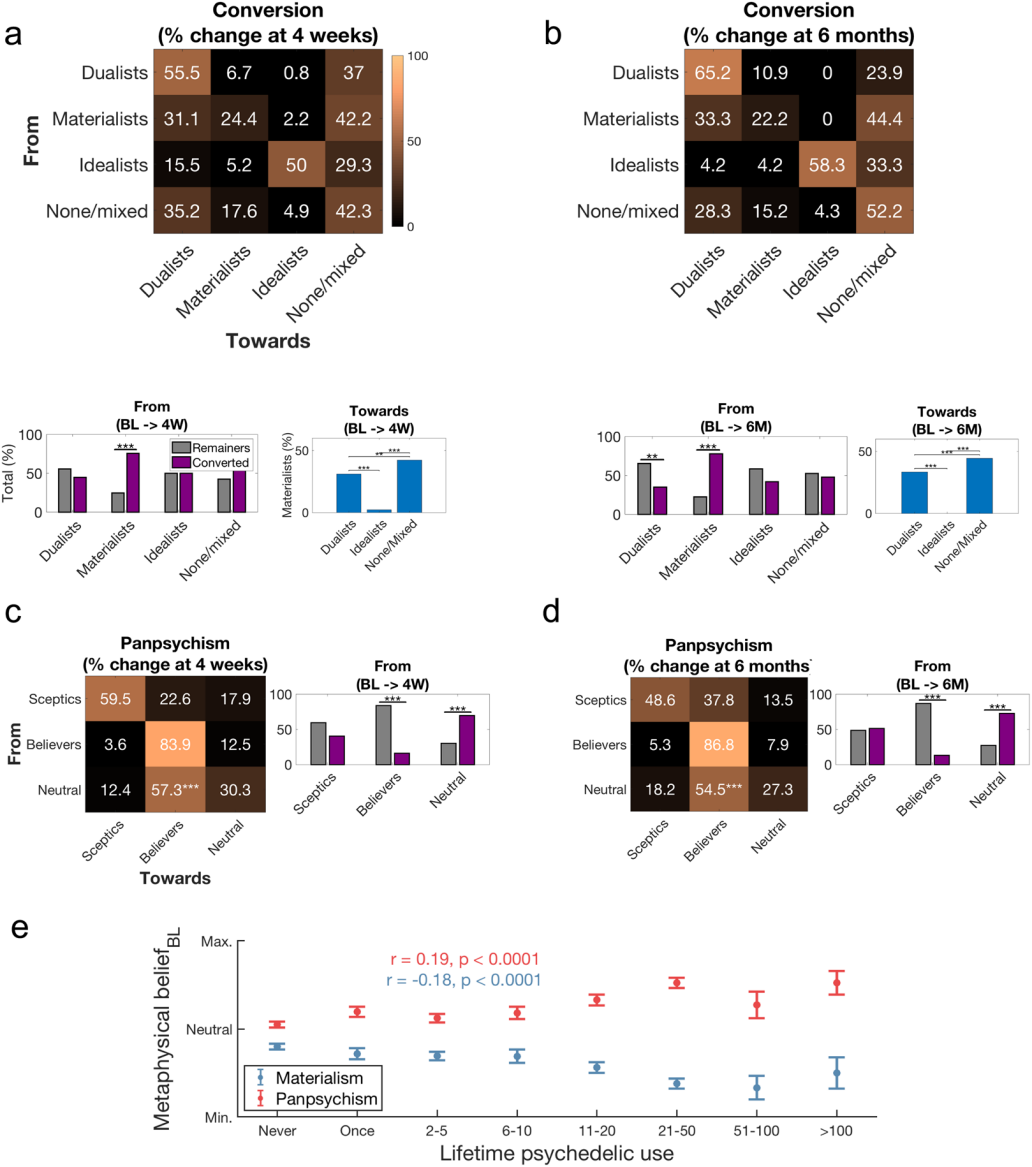
The nature of belief-shifts post-psychedelic-use. Matrices displaying the rate of belief-shift from and towards different ‘hard’ metaphysical positions are displayed at 4 weeks (**a**-above) and 6 months (**b**-above) following the ceremony. Significant rates of change were found only for respondents’ endorsing materialism at 4 weeks (**a**-below) and 6 months (**b**-below), with most of these ‘hard materialists’ leaning towards dualism or equanimity (or reduced hard materialism) post-ceremony. Significant rates of belief-shift were also found for respondents with non-committal views on panpsychism at baseline, who then shifted towards a panpsychist ‘believer’ stance at 4 weeks (**c**) and 6 months (**d**) post-ceremony. (**e**) Lifetime psychedelic use was positively correlated with panpsychist views and negatively correlated with hard materialistic views measured at baseline. (*p < 0.05, **p < 0.01, ***p < 0.001).

To further explore the relationship between psychedelic use and shifts in participant’s metaphysical positions, we separated the sample into four groups corresponding to which metaphysical position participants mostly strongly endorsed at baseline. Respondents with either no positive endorsement or scoring equally high on more than one item were grouped under the label ‘none/mixed’, otherwise they were categorised as either: dualists, idealists or materialists. Results showed that baseline ‘hard-materialists’ tended to shift away from this position after psychedelic use. In fact, such shifts were more common than not. We also found that among those who *did* shift, the nature of the shift was either towards the ‘none/mixed’ position or ‘hard-dualism’. Intriguingly, shifts away from polar metaphysical views to more moderate, ‘softer’ positions, was also evident for a large portion (37%) of baseline ‘hard-dualists’ who tended to reject any preference or endorse an equanimous (i.e. mixed) position post-psychedelic-use (Fig. 2a,b). Separately, however, we observed that those who held more moderate views on panpsychism became more convinced of this position post-psychedelics (labelled ‘believers’) (Fig. 2c,d). These prospective findings were matched by correlations between lifetime psychedelic-use and stronger panpsychist and weaker materialist views, at baseline (Fig. 2e).

### Non-physicalist beliefs and well-being

**Figure 3:**
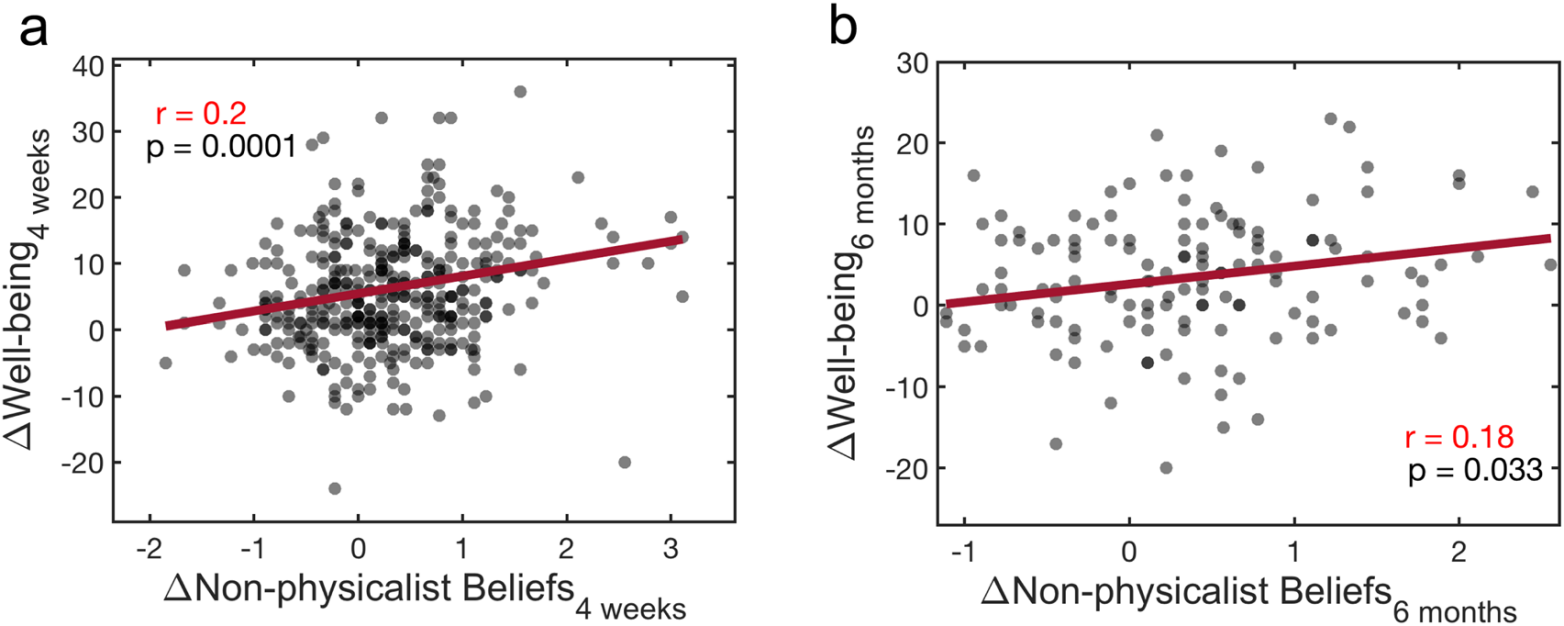
Shifts away from hard materialistic beliefs are associated with increases in well-being. A positive correlation was observed for shifts away from hard materialism versus changes in well-being at both (**a**) 4 weeks and (**b**) 6 months.

A significant positive correlation was found between shifts away from hard-materialism (the NPB factor) and changes in well-being. The correlation was significant at 4 weeks and at 6 months post-ceremony (Fig. 3).

### Process of change modelling

**Figure 4:**
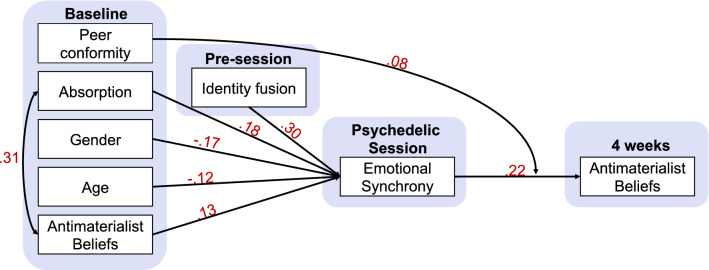
Changes in non-physicalist beliefs are moderated by baseline variables and pre-state identify fusion and mediated by acute emotional synchrony during the psychedelic session. Path model showing changes in Non-physicalist Beliefs to be affected by several demographic and trait characteristics including absorption, gender and age, mediated through perceived emotional synchrony during the psychedelic group session. The effect of synchrony on non-physicalist beliefs was conditional on respondents’ baseline scores of peer conformity. Standardized β-coefficients are shown for significant (p < 0.05) regression paths (not shown are additional significant correlations between non-physicalist beliefs at baseline and absorption with gender, r = 0.19 and r = 0.16, respectively, as well as a significant effect between beliefs at baseline and at 4 weeks post-session; β = 0.75.

A path analysis was performed to examine mechanisms associated with shifts in the relevant non-physicalist beliefs (see “Methods” for details). Included in the model were items and scales pertaining to the acute subjective effects of psychedelics as well as environmental and social-contextual variables relevant to the ceremony experience. Results supported a model with excellent fit (Supplementary Table 2) in which perceived ‘emotional synchrony’ with other participants—moderated by baseline scores of peer conformity—predicted subsequent changes in the NPB factor. Acute emotional synchrony was itself predicted by trait absorption, gender, age, baseline beliefs, plus identity fusion (i.e., identification with the ceremony group) assessed shortly prior to the experience (see “Methods” for details) (Fig. 4).

### Validation with data from a controlled clinical trial

**Figure 5:**
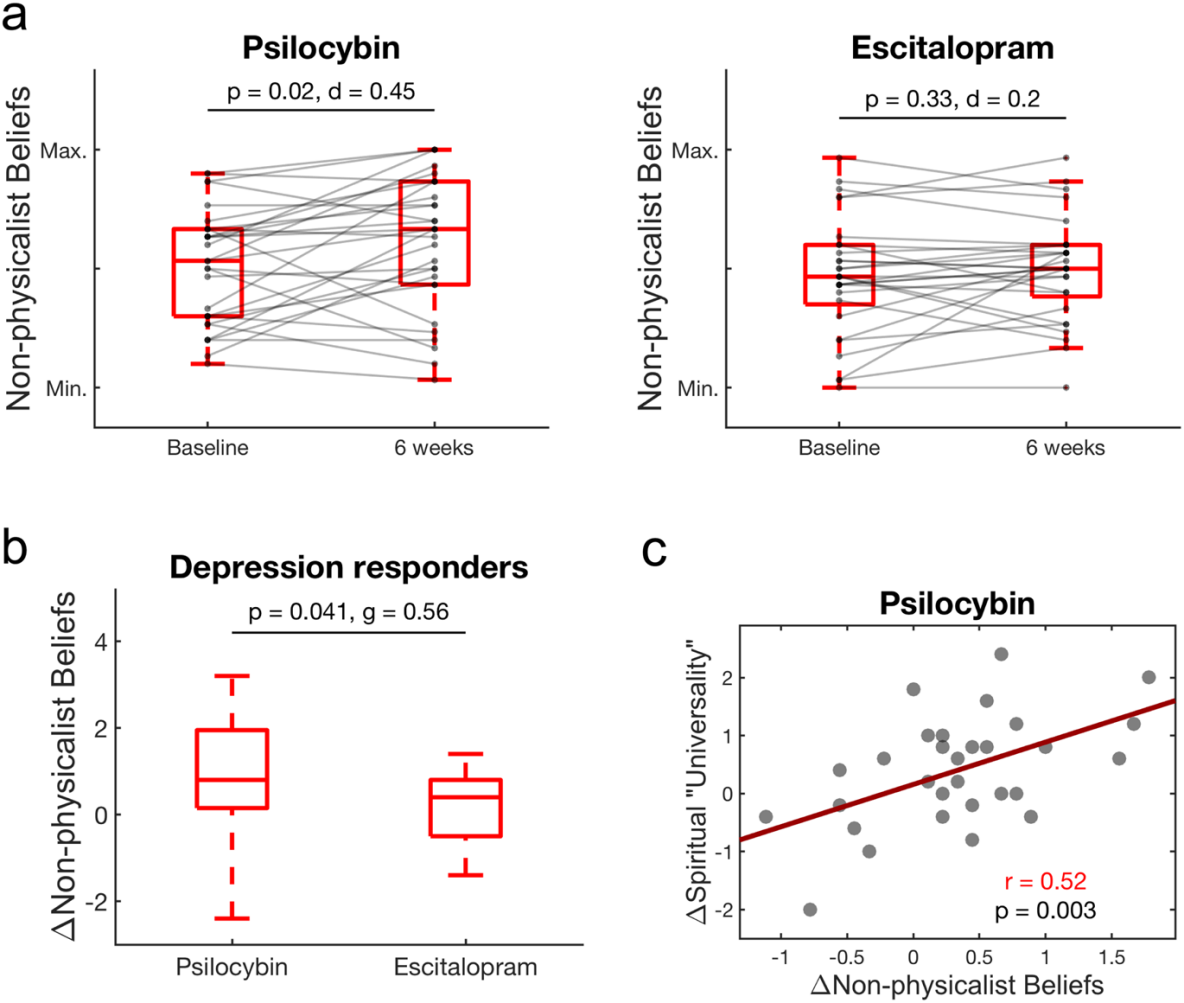
Consistent shifts away from physicalism after psilocybin therapy for depression: (**a**) significant shifts away from hard physicalism were only seen for psilocybin and not the escitalopram condition at the 6 week endpoint versus baseline (Bonferroni-corrected; p values and Cohen’s d effect sizes shown). (**b**) Greater belief-shifts in the predicted direction were found for treatment responders in the psilocybin condition versus responders in the escitalopram group (p value and Hedges’ g effect size shown). (**c**) Shift in non-physicalist beliefs were significantly associated with increases in ‘Spiritual Universality’ (STS scale) at the 6-week endpoint versus baseline, and this was specific for the psilocybin group (i.e., it was not seen in the escitalopram group).

To test the validity and replicability of our findings, we included items corresponding to the NPB in a double-blind randomized-controlled trial comparing a group (*n* = 30) receiving psilocybin therapy with another undergoing a 6-week course of escitalopram (*n* = 29) (See “Methods” for details of trial design). Results replicated well across the independent studies. That is, a significant drug *versus* time (before treatment and 6 weeks after) interaction was observed (*F*(56) = 3.13, *p* = 0.041, one-tailed). More specifically, post-hoc tests reveal that shifts away from hard materialism were evident in the psilocybin group only (Z = 2.28, *p* = 0.02, *d* = 0.45). The escitalopram group showed no changes in NPB (Z = 0.24, *p* = 0.33, *d* = 0.2). (Fig. 5a). Importantly, consistent with the above-reported findings of a relationship between belief shifts and positive mental health outcomes, significantly greater shifts away from hard materialistic beliefs (the NPB factor) were found for those patients who showed a clinically meaningful response to psilocybin only (response is defined as at least 50% reduction in depression scores from baseline to week 6), versus those who showed a response to escitalopram (Z = 1.74, *p* = 0.041, *g* = 0.56, 90% CI [− 0.17, 1.26]) (Fig. 5b). Finally, we found that the belief-shifts in the psilocybin condition were largely correlated with positive endorsement of a unifying spiritual principle (measured at the same timepoints as metaphysical beliefs; see “Supplementary Methods” for the items used), indicating that changes in metaphysical beliefs are related to changes in spiritual beliefs, and are specific to the action of psychedelics versus a conventional antidepressant drug (Fig. 5c).

## Discussion

The present study sought to test the hypothesis that psychedelic experiences mediate changes in metaphysical beliefs concerning the nature of reality, consciousness and ‘fate’. Converging cross-sectional, prospective observational and controlled research data suggest a relationship between psychedelic experiences and shifts away from positions of hard physicalism and towards panpsychism, dualistic, and fatalistic beliefs. The observed changes were enduring, persisting for up to 6 months in most domains, with the exception of fatalistic determinism. Moreover, the large-sample prospective/observational and smaller but well-controlled research sample findings converged, implying that psychedelic-use may be a key casual determinant of the relevant shifts in metaphysical beliefs. Furthermore, the belief-shifts were correlated with positive mental health changes; namely, improvements in well-being in the observational data and depression scores in the controlled research data.

Included within this study results was the specific finding of an increase in fatalistic determinism following ceremonial psychedelic use. Unlike non-physicalist beliefs, these changes were not sustained in time. One possible explanation for this discrepancy may be due to the necessity of culturally or institutionally-mediated reinforcement to consolidate these changes, e.g., through religion or a socio-political milieu. In this specific case, it is plausible that non-physicalist beliefs feature more heavily in ‘psychedelic culture’, compared with beliefs concerning fatalistic determinism (see popular literary and artistic examples here^24,48^), and therefore the former beliefs are more exposed to reinforcing influences than the latter. This is in line with the notion that the context may have an enduring influence on these lasting effects^41,42,45^.

Path analyses on the psychedelic ceremony-derived data highlighted the predictive relevance of certain psychological traits, including ‘absorption’—which indexes differential proclivity to states of immersion or ‘flow’^49^, and peer conformity. Trait absorption has been found to predict propensity for spiritual-type experiences^50^ and is related to trait suggestibility^49^—as well as a serotonin 2A receptor genetic polymorphism^51^—the key receptor target for classic psychedelics^52^. Peer conformity has also been found to relate to suggestibility^53^.

These findings extend current understandings of the interaction between the psychedelic state and both suggestibility and trait absorption^40,54,55^, by highlighting how changes in metaphysical beliefs may become prominent in group settings during psychedelic-use. These relationships evoke historical cases where psychedelics have been exploited for purposes of mind-control^56^. Important ethical considerations are raised by the prospect of pharmacologically catalysed changes in core beliefs occurring without prior informed consent. In light of these findings, future studies and services that offer psychedelic experiences may be ethically obliged to include reference to the possibility of belief change as part of informed consent procedures. Relatedly, further research is critical to test the robustness and reliability of the phenomenon, as this will determine how important it is that the information be provided to research participants and service users.

We are of the view that psychedelic therapy depends on an interaction between a biological action of the drug and non-pharmacological contextual factors^45^. Just as the management of context is essential for therapeutic outcomes, so it may require careful and responsible management with regard to other psychological outcomes such as potential changes in metaphysical beliefs. For relevant discussions see Timmermann et al.^45^, Carhart-Harris and Friston^41^, and Letheby^57^.

Regarding demographic variables, age and gender were other relevant predictors of belief shifts: specifically, lower age and female gender were predictive of the relevant changes. The relationship between lower age and suggestibility is well established^58^. Pre-state identity fusion (feelings of identification with the ceremony group^59^) was another relevant variable moderating susceptibility to the relevant belief-shifts. All of these variables moderated ‘emotional synchrony’^60^ felt during the psychedelic ceremony itself. Higher emotional synchrony scores strongly mediated the relevant shifts in metaphysical beliefs, i.e., away from physicalism.

The path modelling results add further weight to the principle that outcomes of psychedelic-use strongly depend on contextual variables^61^ and mesh with other work suggesting that ritual can enhance cultural transmission^62^. It is logical to surmise that the influence of cultural context, and ritual in particular, could be further enhanced via the pro-suggestibility action of psychedelics—an action that may relate to evidence for the neuroplasticity-enhancing effects of psychedelics^63^. Advancing our understanding of the biological aspect of psychological transformations is a research area deserving special focus. For example, the relationship between serotonin 2A receptor agonism-induced cortical plasticity seems especially relevant in this regard^15,63^.

A recent hierarchical predictive coding inspired model of the brain action of psychedelics, known as ‘REBUS’ (RElaxed Beliefs Under pSychedelics), may provide some useful inspiration for aiding investigations of the neurobiology of belief change processes^41^. The REBUS model proposes that rendering high-level beliefs and assumptions more plastic (by reducing the precision-weighting on these prior beliefs) under psychedelics is a key mechanism underlying their acute phenomenological and potential therapeutic effects. Like previous work in other psychological domains (discussed above), our results indicate that changes in beliefs tend to occur in a particular direction (i.e., in the present case, away from physicalism). At the aggregate level, the dominant direction of this shift points away from ‘hard’ metaphysical views, and away from ‘hard materialism’ in particular. However, results also reveal signs of a ‘hardening’ (or increase in confidence or precision-weighting) in beliefs in panpsychism and related domains of ‘hard’ dualism. Changes in confidence in beliefs under psychedelics may be dose-sensitive^64^, and confidence (i.e., precision-weighting) or the *content* of actual beliefs are likely to be sensitive to indirect influences—beyond drug action alone^65^. More research is needed to better examine the usefulness of different theoretical models for explaining the findings we present here.

It important to highlight that the data we present here corresponds to hundreds of responses from participants taking part in a range of ceremonial contexts, supplemented by data from rigorously controlled clinical research. Our findings could, therefore, be interpreted as suggesting a degree of *context-independency* in the specific nature of the observed changes. It is relevant to consider however, that even in therapeutic and research environments, the psychedelic experience and surrounding process will be imbued with cultural influences that could easily affect beliefs in a particular direction^45^.

More work is needed to address the question of whether the apparent directional nature of post-psychedelic belief-shifts are context independent or otherwise. Aside from assessing more diverse contexts, one might also consider measuring the metaphysical beliefs of others attending and organising ceremonies, as well as those managing and participating in clinical research, to see if there is any evidence of inter-personal or cultural transmission of beliefs. It may transpire that the two possibilities (context dependency versus independency) are not mutually exclusive, e.g., acute belief *relaxation* may be somewhat context independent but sub-acute belief *revision* and consolidation may be more dependent on context. That self-organized or autopoietic processes are involved in any putative direction-specific, context-independent psychological changes seen after psychedelic-use is a plausible possibility also deserving of further thought and research. Addressing this question will not be trivial but may also bear relevance to the related question of whether psychedelics can be ‘intrinsically’ therapeutic (i.e., that context-independent mechanisms driving the therapeutic potential of psychedelics relate to their capacity to shift metaphysical beliefs in a specific direction).

Accounts of the apparent modulation of core beliefs via psychedelic drug use appears to be consistent across different Western cultures^44^, and the types of beliefs that participants appear to gravitate towards (e.g., panpsychism) after psychedelic use are somewhat consistent with those that are culturally held by many indigenous and mestizo populations (e.g., worldviews related to animism^48,66^), as well as some “New Age” groups^67^. One could interpret these observations as consistent with a context-independent belief shift, or alternatively, evidence of a bidirectional causal relationship between cultural values and psychedelic use, e.g., that certain cultures and viewpoints lend themselves toward psychedelic use, although the availability of psychedelic plants will be a key factor. One way to test the causality question might be to assess metaphysical beliefs in populations with limited access to psychedelic plants or drugs. Another approach would be to assess whether an increase in the prevalence of psychedelic drug use e.g., as in happening presently in certain Western countries^68^, is translating into shifting metaphysical beliefs.

Assessing the value of competing philosophical positions is beyond the remit of the present work—and, some might argue, beyond the remit of science. However, we acknowledge that this matter has important ethical, social and psychological implications and therefore merits some further comment. It would be hasty, in our view, to interpret this study’s findings as evidence in favour of the positive value of e.g., non-physicalist, pro-panpsychist or fatalistic beliefs. For example, the adoption of non-physicalist or supernatural beliefs have been associated with maladaptive coping strategies such as avoidance or escapism, e.g. via ‘spiritual bypassing’^69–72^. One pragmatic way in which science could approach this issue would be to assess how different metaphysical positions interface with individual (mental) health, as well as other indices of individual plus group or societal health, e.g., population-level well-being, as well as behaviours linked to ecological health.

The present study found a positive association between changes in metaphysical beliefs away from physicalism and increases in psychological well-being. Moreover, this finding was replicated in independent data from controlled research where belief-shifts in a consistent direction were found in responders (vs. non-responders) to psilocybin-therapy for depression, and no such relationship was evident for an active-treatment (selective serotonin reuptake inhibitor, antidepressant, ‘escitalopram’) control arm, even in those who responded to that intervention. These findings suggest that non-physicalist beliefs may be psychologically protective during psychological distress, resonant with the hypothesis that religious practices and beliefs serve a similar function^73,74^. An alternative explanation might be that it is the relaxation of—e.g., too-heavily-weighted, metaphysical beliefs—and not the specific shift away from physicalism, which may be related to these changes. Further work is needed to test these, and other, possibilities.

It is important to note some limitations to our findings. We did not collect respondents’ and patients’ religious affiliations at baseline, differential environmental susceptibilities, or interests concerning metaphysical beliefs. It is plausible therefore that we recruited an atypical sample skewed towards individuals who were sensitive to the belief shifts observed here. In this regard, it may be noteworthy that both samples were largely comprised of Western, well-educated people.

Although we replicated our findings in two different groups, significant differences exist between both samples (e.g., the number of respondents, levels of psychological distress, corresponding expectancies etc.). Our failure to measure the cultural milieu for each population, including the views of those surrounding the respondent or patient at the time of psychedelic-use and afterwards, is an oversight. It is plausible, for example, that somewhat consistent non-physicalist views were held by co-ceremony attendees and the research staff in the clinical trial, which transferred over to the participants in question. Such transference could also occur due an influence from the broader cultural milieu associated with psychedelic-use, e.g., influencing how the participants interpreted or made sense of their psychedelic experiences.

These caveats aside, it remains of interest that the present study discovered converging evidence in support of a potential causal influence of psychedelic-use on consistent changes in metaphysical beliefs. These lines of evidence include: (1) that the belief-shifts were observed prospectively (i.e. before vs after psychedelic-use) in a large sample, (2) especially pronounced shifts were seen in psychedelic-naïve participants, (3) the quality of the acute experience (i.e., emotional synchrony) was found to be a significant mediator of the belief-shifts, (4) extent of past psychedelic-use correlated with beliefs in the predicted direction, and (5) independent data from a separate controlled study in a different population, replicated our observational research findings. It is also relevant that previous controlled studies have demonstrated post-psychedelic changes in other psychological domains after controlling for potential confounding factors^29,75,76^.

To conclude, this mixed method study, comprising of an observational plus controlled research design, has assessed the relationship between psychedelic drug use and metaphysical beliefs. Findings are consistent with the inference that psychedelic-use inclines individuals away from hard physicalist beliefs and towards dualistic, panpsychist, and fatalistic beliefs, thus highlighting the potential of psychedelics to alter some of the most deep-seated and influential human beliefs. These results have profound scientific, societal, political and philosophical implications, and therefore demand further investigation.

## Methods

### Design and participants

Respondents planning to attend a ceremony involving a psychedelic substance (psilocybin/magic mushrooms/truffles, ayahuasca, DMT, San Pedro, LSD/1P‐LSD) were recruited via online advertisements and invited to sign up for the study via the website www.psychedelicsurvey.com. Subjects were offered to sign up if planning to attend in a ceremony involving the use of a plant medicine (for example within an Ayahuasca retreat) or another guided psychedelic experience. Eligibility criteria to participate consisted in: being 18 years or older, good comprehension of the English language, and the intention to participate in a retreat, ceremony or other guided experience involving a classic psychedelic (i.e., 5-HT2A agonist such as psilocybin, DMT, mescaline or LSD). The study received a favourable opinion from the Imperial College Research Ethics Committee and was sponsored by the Imperial Joint Research and Compliance Office. All participants provided informed consent. Automatic email reminders were then sent out at multiple time‐points at baseline (one week before the experience), one day after the experience and 4 weeks after the indicated date of the experience to participants who signalled consent, including links to surveys hosted on the online platform SurveyGizmo. All methods were carried out by relevant guidelines and regulations (see Kettner et al.^43^ and Table 2 for study details and sample characteristics at baseline).

**Table 2:** Demographic information collected at baseline^43^.

Total N	819
Age	44.4 ± 12.6
**Gender**	
Female	359 (43.8%)
Male	455 (55.6%)
Other	5 (0.6%)
**Nationality**	
United States	359 (43.8%)
United Kingdom	160 (19.5%)
Australia	31 (3.8%)
Germany	28 (3.4%)
Canada	26 (3.2%)
Other countries (53 in total)	215 (26.3%)
**Education**	
None	6 (0.7%)
High School or equivalent (GED)	62 (7.6%)
Associate/Technical Degree	58 (7.1%)
College Diploma	250 (30.1%)
Master’s Degree	275 (33.6%)
Doctorate or Professional Degree	168 (20.5%)
**Employment**	
Student	46 (5.6%)
Working full-time	520 (63.4%)
Working part-time	120 (14.7%)
Retired	73 (8.9%)
Unemployed	60 (7.3%)
Median household income	9000 $/month
**Ethnicity**	
White	743 (90.7%)
Black or African American	12 (1.5%)
Asian	48 (5.9%)
American Indian or Alaska native	3 (0.4%)
Unknown/prefer not to say	11 (1.3%)/23 (2.8%)
**Marital status**	
Cohabiting with partner	101 (12.3%)
Married	340 (41.5%)
Divorced	86 (10.5%)
Separated	29 (3.5%)
Never married	254 (31.0%)
Widowed	9 (1.1%)

Survey findings were complemented by results from controlled a controlled clinical-trial comparing psilocybin versus escitalopram treatment for major depression. The data from 59 participants with major depression (> 17 on Hamilton-Depression [HAM-D-17] scale at screening) who completed the trial was used (30 participants for psilocybin and 29 for escitalopram). Participants ranging from 18 to 80 years old were recruited formally through trial networks, informally via social media, and through other sources (see Carhart-Harris et al.^77^ for demographic information). Main exclusion criteria consisted in immediate family or personal history of psychosis, medically significant health conditions (assessed by a physician), a history of serious suicide attempts, positive pregnancy test, contraindications to undergo an MRI or taking selective serotonin-reuptake inhibitors (SSRI’s), previous use of escitalopram or a pre-existing condition that could jeopardize the rapport between the participant and the trial mental health caregivers. Participants received either two doses of an active dose of psilocybin (25 mg) and 6 weeks of daily placebo (psilocybin group) or two doses of a control dose of psilocybin (1 mg) and daily escitalopram (escitalopram group). Participants completed an adapted version of the Metaphysical Beliefs Questionnaire (see “Measures”) at baseline and at 6 weeks following the start of treatment, the latter being the key endpoint of the trial. Home Office Schedule 1 Drug Licenses, UK Medicines & Healthcare products Regulatory Agency, and GDPR approvals were obtained. All study protocols were approved by the Brent Research Ethics Committee, the Health Research Authority, Imperial College London and the Joint Research Compliance office. All methods were carried out by relevant guidelines and regulations (see Carhart-Harris et al.^77^ for details regarding the study protocol and the main results of the trial). (ClinicalTrials.gov Identifier: NCT03429075, registered on February 12, 2018; EudraCT: 2017-000219-18).

### Measures

There were two main outcome measures in this study which were employed at baseline (one week prior to the experience), 4 weeks, and 6 months after attending the ceremony. They were (1) a self-constructed *Metaphysical Beliefs Questionnaire* (MBQ; see Table 1), and (2) items extracted from the Free Will and Fatalistic Determinism subscales (containing nine items in total) of the FAD-Plus questionnaire, a validated measure of lay views on free will and determinism^4^ (see “Supplementary Methods” for the items used).

The MBQ consists of 13 items: three directly assessing beliefs concerning the mental and the physical (i.e. beliefs in physicalism/materialism, idealism and dualism)—that were directly extracted from previous work^6^, and ten that were conceived based on a review of literature pertaining to psychedelic-induced changes in metaphysical beliefs^23–25,78^, qualitative studies^79–81^, and transcribed interviews performed in our own research involving DMT and psilocybin administrations to healthy volunteers^82,83^. Respondents were given the following instructions: ‘You will be presented with a series of statements about the nature of consciousness and reality. Please read each statement carefully and rate the extent to which you agree or disagree with each item using the following scale’.

The relevant ten items were conceived with the intention of assessing beliefs in separate domains, including panpsychism, idealistic/solipsistic beliefs (i.e. ‘*the physical world is an illusion generated by the mind*’), beliefs in the literal transcendence of the constraints of this universe, and related beliefs in the existence of other worlds or universes that one can visit, monism (i.e. the position that there is only one fundamental type of substance), idealism (i.e. that reality is entirely constituted by mind or consciousness), physicalism or materialism (i.e. the belief that reality is entirely constituted by physical or material objects and processes), the notion that all mental events (including the sense of self) can be traced back to brain activity and more nuanced positions highlighting the embeddedness of conscious experience in brain, body and world (i.e. enactivism^84^).

Additionally, we explored the acute experiences of ceremony participants by issuing questionnaires enquiring about this one day after the experience itself. The Mystical Experience Questionnaire (MEQ^21^), visual scales derived from the 11-dimension Altered States of Consciousness Questionnaire (ASC^85^) and Perceived Emotional Synchrony Scale (PESC^86^) were measured in relation to the acute psychedelic experience. Additionally, the personality trait Absorption^49^ (known to be a contextual factor which can strongly predict the character of psychedelic experiences^54^) and the subscale *peer conformity* from the Multidimensional Iowa Suggestibility Scale^53^ (suggestibility is known to be sensitive to psychedelic administration^40^) were both measured at baseline. Finally, identity fusion (the visceral experience of ‘oneness’ with the group) was measured shortly before the session as a single pictographic item^59^.

We also explored the extent to which any changes in beliefs might be associated with changes in psychological well-being. Well-being was measured at baseline, 4 weeks after, and 6 months after the ceremony using the Warwick–Edinburgh Mental Wellbeing Scale^87^, which has been shown to be sensitive to the effects of psychedelics^55,76,88^.

For controlled research, a shortened version of the Metaphysical Beliefs Questionnaire was used (containing the following items: Ontological Transcendentalism, Supernatural Transcendentalism, Dualism, Materialism, and Non-naturalism), from which the NPB factor was extracted. Also, for the controlled study, the ‘Universality’ subscale from the Spiritual Transcendence Scale^89^ (see “Supplementary Methods” for the items) was used to determine the association between changes in beliefs and changes in spirituality.

### Statistical analysis

An exploratory factor analysis was performed on the MBQ (Table 1) at baseline. Multiple heuristics were used to determine the optimal number of factors to retain among the 13 items included in the analysis^90^. Parallel analysis, optimal coordinate and the ‘Kaiser rule’^91^, suggested a three-factorial solution, whereas acceleration factor and visual examination of the scree plot a unifactorial solution (Supplementary Fig. 4). Examination of factor loadings showed that in the three-factorial solution, only one and two items loaded on factors two and three, respectively. Thus, a unifactorial solution was chosen, with standardized loadings presented in Table 1. After removal of items with a. factor loading < 0.4^92^. Finally, Cronbach’s alpha was used to determine internal consistency.

Analyses were conducted to determine changes in beliefs (baseline vs 4 week post ceremony, and baseline vs 6 months post ceremony) for all respondents. Separate analyses were performed using responses from participants with no previous psychedelic experiences, on the grounds that respondents with previous psychedelic experiences might already have had shifts in the relevant beliefs. Paired t-tests were performed to assess the statistical significance of changes in specific beliefs (the principal factor extracted from the MBQ and the Free-Will and Fatalistic Determinism subscales from the FAD scale). Two-tailed and Bonferroni-corrected p-values are reported. Cohen’s *d* effect sizes are reported for all paired analyses.

Following previous studies^6^, we also performed analyses to determine the rate of conversion from baseline ‘hard’ metaphysical positions regarding materialism, idealism and dualism. In order to do this, we grouped respondents based on their tendency to endorse materialism, dualism, idealism or ‘none/mixed’ based on their initial Likert responses for items 3, 4 and 5 of the MBQ (see Table 1). The single highest score between the three items corresponded to the group each respondent would be allocated. If there were competing highest scores, or if the highest score was below or equal to a neutral score, then subjects were grouped as None/Mixed. Determining the rate of conversion from a hard metaphysical position was done via Chi-squared tests comparing percentages of ‘Remainers’ (those respondents who did not shift between groups in subsequent timepoints) and ‘Converted’ (those who did in any direction). Determining differences between the groups toward which Materialists respondents converted (the only group showing a significant rate of conversion) was also done by performing paired Chi-squared tests for each pair of comparisons (p values were Bonferroni-corrected for multiple comparisons). We also performed a similar procedure using a single item corresponding to panpsychism from the MBQ, by grouping respondents as ‘Believers’, ‘Sceptics’ and ‘Neutral’ according to scores above, below or corresponding to, the neutral Likert score. Chi-Squared tests were performed for comparing Conversion from and towards a different metaphysical position.

A longitudinal exploratory path analysis was performed to determine the psychological mechanisms associated with changes in NPB. Included were items and scales pertaining to the acute subjective effects of the psychedelic as well as environmental and social contextual variables relevant to the ceremony experience. Specifically, the intensity of visual (simple and complex imagery scales; ASC), mystical-type (MEQ) and emotional synchrony experiences (PESC), measured on the day post-session, were expected to predict baseline-corrected NPB scores 4 weeks post-session, although due to non-significance of effects, only the PESC was retained in the model. In the case of collective emotional synchrony experiences, this effect was expected to be moderated by peer conformity measured at baseline, calculated as the sum of the four peer conformity-items from the short suggestibility scale (SSS). Demographic variables, trait absorption (MODTAS), baseline supernatural NPB, and alignment with the group assessed hours before the session via a pictographic identity fusion scale^59^ were included in the model as predictors of acute experience (MEQ and PESC) scores. Whenever a respondent attended more than one psychedelic ceremony and provided multiple reports, only the largest score for each of these scales was considered.

The association between changes in NPB and mental health outcomes was assessed by Pearson-Point correlational analyses between changes in the NPB factor and changes in well-being (changes in WEBMWS scores).

For the controlled research analysis, changes in NPB between baseline and the 6-weeks timepoint was compared between the psilocybin and escitalopram groups using a repeated measures ANCOVA, adjusted for baseline. Post-hoc tests comparing baseline and 6-weeks post-treatment were performed using Wilcoxon signed rank test for dependent samples (Bonferroni-corrected). Changes in NPB Beliefs were compared for psilocybin versus escitalopram remitters using Wilcoxon rank sum test for independent samples. Finally, the association between Changes in NPB and changes in Spiritual Beliefs were assessed using the Universality subscale from the Spiritual Transcendence Scale^89^. Single-sided *p*-values are reported for controlled research analysis as clear hypotheses were derived from the main survey data presented here. Cohen’s *d* and Hedges’ *g* effect sizes are reported for paired and independent tests, respectively.

## Supplementary Information

Supplementary Information.

## Data Availability

Data is available upon reasonable request to the corresponding author.

## References

[1] van Inwagen, P. & Sullivan, M. Metaphysics. *The Stanford Encyclopedia of Philosophy* (2018). https://plato.stanford.edu/archives/spr2018/entries/metaphysics/. Accessed 4 January 2020.

[2] Forstmann M, Burgmer P (2014). Adults are intuitive mind–body dualists. J. Exp. Psychol. Gen..

[3] Stanovich KE (1989). Implicit philosophies of mind: The dualism scale and its relation to religiosity and belief in extrasensory perception. J. Psychol..

[4] Paulhus DL, Carey JM (2011). The FAD-plus: Measuring lay beliefs regarding free will and related constructs. J. Pers. Assess..

[5] Demertzi A (2009). Dualism persists in the science of mind. Ann. N.Y. Acad. Sci..

[6] Reggia JA, Huang D-W, Katz G (2015). Beliefs concerning the nature of consciousness. J. Conscious. Stud..

[7] Bering JM (2006). The folk psychology of souls. Behav. Brain Sci..

[8] Forstmann M, Burgmer P, Mussweiler T (2012). “The mind is willing, but the flesh is weak”: The effects of mind-body dualism on health behavior. Psychol. Sci..

[9] Vohs KD, Schooler JW (2008). The value of believing in free will: Encouraging a belief in determinism increases cheating. Psychol. Sci..

[10] Baumeister RF, Masicampo EJ, Dewall CN (2009). Prosocial benefits of feeling free: Disbelief in free will increases aggression and reduces helpfulness. Personal. Soc. Psychol. Bull..

[11] Aarts H, van den Bos K (2011). On the foundations of beliefs in free will: Intentional binding and unconscious priming in self-agency. Psychol. Sci..

[12] O’Connor, T. & Franklin, C. Free Will. *The Stanford Encyclopedia of Philosophy*. https://plato.stanford.edu/archives/spr2021/entries/freewill/ (2019). Accessed 4 January 2020.

[13] Flanagan O, Caruso GD (2018). Neuroexistentialism: meaning, morals, and purpose in the age of neuroscience.

[14] Miller WR (2004). The phenomenon of quantum change. J. Clin. Psychol..

[15] Brouwer A, Carhart-Harris RL (2020). Pivotal mental states. J. Psychopharmacol..

[16] Greyson B (1983). The near-death experience scale. Construction, reliability, and validity. J. Nervous Mental Disease.

[17] Josipovic Z (2010). Duality and nonduality in meditation research. Conscious. Cogn..

[18] Lynn SJ, Evans J (2017). Hypnotic suggestion produces mystical-type experiences in the laboratory: A demonstration proof. Psychol. Conscious. Theory Res. Pract..

[19] White F (1987). The Overview Effect: Space Exploration and Human Evolution.

[20] Edmondson D (2011). From shattered assumptions to weakened worldviews: Trauma symptoms signal anxiety buffer disruption. J. Loss Trauma.

[21] Barrett FS, Johnson MW, Griffiths RR (2015). Validation of the revised Mystical Experience Questionnaire in experimental sessions with psilocybin. J. Psychopharmacol..

[22] Pahnke WN, Richards WA (1970). Implications of LSD and experimental mysticism. J. Psychoactive Drugs.

[23] Grof S (1975). Realms of the Human Unconscious.

[24] Strassman R (2001). DMT: The Spirit Molecule.

[25] Shanon B (2005). The Antipodes of the Mind: Charting the Phenomenology of the Ayahuasca Experience.

[26] Davis AK (2020). Survey of entity encounter experiences occasioned by inhaled *N*,*N*-dimethyltryptamine: Phenomenology, interpretation, and enduring effects. J. Psychopharmacol..

[27] Griffiths RR, Richards WA, Johnson MW, McCann UD, Jesse R (2008). Mystical-type experiences occasioned by psilocybin mediate the attribution of personal meaning and spiritual significance 14 months later. J. Psychopharmacol..

[28] Kometer M, Pokorny T, Seifritz E, Volleinweider FX (2015). Psilocybin-induced spiritual experiences and insightfulness are associated with synchronization of neuronal oscillations. Psychopharmacology.

[29] Griffiths RR, Hurwitz ES, Davis AK, Johnson MW, Jesse R (2019). Survey of subjective ‘God encounter experiences’: Comparisons among naturally occurring experiences and those occasioned by the classic psychedelics psilocybin, LSD, ayahuasca, or DMT. PLoS ONE.

[30] Ross S (2016). Rapid and sustained symptom reduction following psilocybin treatment for anxiety and depression in patients with life-threatening cancer: A randomized controlled trial. J. Psychopharmacol..

[31] Forstmann M, Yudkin DA, Prosser AMB, Heller SM, Crockett MJ (2020). Transformative experience and social connectedness mediate the mood-enhancing effects of psychedelic use in naturalistic settings. Proc. Natl. Acad. Sci..

[32] Maslow AH (1959). Cognition of being in the peak experiences. J. Genet. Psychol..

[33] Stace W (1960). Mysticism and Philosophy.

[34] Richards WA, Rhead JC, DiLeo FB, Yensen R, Kurland AA (1977). The peak experience variable in DPT-assisted psychotherapy with cancer patients. J. Psychedelic Drugs.

[35] Shiota MN, Keltner D, Mossman A (2007). The nature of awe: Elicitors, appraisals, and effects on self-concept. Cogn. Emot..

[36] Yaden DB (2016). The overview effect: Awe and self-transcendent experience in space flight. Psychol. Conscious. Theory Res. Pract..

[37] Hanley AW, Nakamura Y, Garland EL (2018). The Nondual Awareness Dimensional Assessment (NADA): New tools to assess nondual traits and states of consciousness occurring within and beyond the context of meditation. Psychol. Assess..

[38] Wikitionary. Ontological shock. https://en.wiktionary.org/wiki/ontological_shock (2021). Accessed 6 February 2021.

[39] Miller WR, C’Dde Baca J (2001). Quantum Change: When Epiphanies and Sudden Insights Transform Ordinary Lives.

[40] Carhart-Harris RL (2015). LSD enhances suggestibility in healthy volunteers. Psychopharmacology.

[41] Carhart-Harris RL, Friston KJ (2019). REBUS and the anarchic brain: Toward a unified model of the brain action of psychedelics. Pharmacol. Rev..

[42] Dupuis D (2021). The socialization of hallucinations. Cultural priors, social interactions and contextual factors in the use of ayahuasca. Transcult. Psychiatry..

[43] Kettner H (2021). Psychedelic communitas: Intersubjective experience during psychedelic group sessions predicts enduring changes in psychological wellbeing and social connectedness. Front. Pharmacol..

[44] Lerner M, Lyvers M (2006). Values and beliefs of psychedelic drug users: A cross-cultural study. J. Psychoactive Drugs.

[45] Timmermann C, Watts R, Dupuis D (2020). Towards psychedelic apprenticeship: Developing a gentle touch for the mediation and validation of psychedelic-induced insights and revelations. Transcult. Psychiatry.

[46] Johnson, M. W. & Yaden, D. B. There’s no good evidence that psychedelics can change your politics or religion. *Scientific American*. https://www.scientificamerican.com/article/theres-no-good-evidence-that-psychedelics-can-change-your-politics-or-religion/ (2020). Accessed 6 February 2021.

[47] Letheby, C. *The philosophy of psychedelic transformation*. Doctoral Dissertation. University of Adelaide (2016).

[48] Luna LE, Amaringo P (1999). Ayahuasca Visions: The Religious Iconography of a Peruvian Shaman.

[49] Jamieson GA (2005). The modified Tellegen Absorption Scale: A clearer window on the structure and meaning of absorption. Aust. J. Clin. Exp. Hypn..

[50] Lifshitz M, van Elk M, Luhrmann TM (2019). Absorption and spiritual experience: A review of evidence and potential mechanisms. Conscious. Cogn..

[51] Ott U, Reuter M, Hennig J, Vaitl D (2005). Evidence for a common biological basis of the absorption trait, hallucinogen effects, and positive symptoms: Epistasis between 5-HT2a and COMT polymorphisms. Am. J. Med. Genet. Neuropsychiatr. Genet..

[52] Nichols DE (2016). Psychedelics. Pharmacol. Rev..

[53] Kotov, R., Bellman, S. B. & Watson, D. B. Multidimensional Iowa suggestibility scale (MISS) bried manual. *Soneybrook Med. Website* (2004).

[54] Studerus E, Gamma A, Kometer M, Vollenweider FX (2012). Prediction of psilocybin response in healthy volunteers. PLoS One.

[55] Haijen ECHM (2018). Predicting responses to psychedelics: A prospective study. Front. Pharmacol..

[56] Lee M, Shlain B (1985). Acid Dreams: The Complete Social History of LSD.

[57] Letheby C (2016). The epistemic innocence of psychedelic states. Conscious. Cogn..

[58] Mitchell KJ, Johnson MK, Mather M (2003). Source monitoring and suggestibility to misinformation: Adult age-related differences. Appl. Cogn. Psychol..

[59] Swann WB, Gómez Á, Seyle DC, Morales JF, Huici C (2009). Identity fusion: The interplay of personal and social identities in extreme group behavior. J. Pers. Soc. Psychol..

[60] Páez D, Rimé B, Basabe N, Wlodarczyk A, Zumeta L (2015). Psychosocial effects of perceived emotional synchrony in collective gatherings. J. Pers. Soc. Psychol..

[61] Carhart-Harris R (2018). Psychedelics and the essential importance of context. J. Psychopharmacol..

[62] Whitehouse H (2004). Modes of Religiosity: A Cognitive Theory of Religious Transmission.

[63] Ly C (2018). Psychedelics promote structural and functional neural plasticity. Cell Rep..

[64] Safron A (2021). On the varieties of conscious experiences: Altered beliefs under psychedelics (ALBUS). PsyArxiv.

[65] McGovern HT, Leptourgos P, Hutchinson BT, Corlett PR (2021). Do psychedelics change beliefs?. PsyArxiv.

[66] Dobkin De Rios M (1984). The vidente phenomenon in third world traditional healing: An amazonian example. Med. Anthropol..

[67] Hanegraaf WJ (1995). New Age Religion and Western Culture. Esotericism in the Mirror of Secular Thought.

[68] Yockey RA, Vidourek RA, King KA (2020). Trends in LSD use among US adults: 2015–2018. Drug Alcohol Depend..

[69] Welwood J (2002). Toward a Psychology of Spiritual Awakening.

[70] Masters RA (2010). Spiritual Bypassing.

[71] Kornfield J (2000). After the Ecstasy, the Laundry.

[72] Hayes SC (2019). A Liberated Mind.

[73] Rosmarin DH, Krumrei EJ, Andersson G (2009). Religion as a predictor of psychological distress in two religious communities. Cogn. Behav. Ther..

[74] Jarvis GE, Kirmayer LJ, Weinfeld M, Lasry JC (2005). Religious practice and psychological distress: The importance of gender, ethnicity and immigrant status. Transcult. Psychiatry.

[75] Forstmann M, Sagioglou C (2017). Lifetime experience with (classic) psychedelics predicts pro-environmental behavior through an increase in nature relatedness. J. Psychopharmacol..

[76] Kettner H, Gandy S, Haijen ECHM, Harris RLC (2019). From egoism to ecoism: Psychedelics increase nature relatedness in a state—Mediated and context—Dependent manner. J. Environ. Res. Public Heal..

[77] Carhart-Harris R (2021). Trial of psilocybin versus escitalopram for depression. N. Engl. J. Med..

[78] Shanon B (2010). The epistemics of ayahuasca visions. Phenomenol. Cogn. Sci..

[79] Watts R, Day C, Krzanowski J, Nutt D, Carhart-Harris R (2017). Patients’ accounts of increased “connectedness” and “acceptance” after psilocybin for treatment-resistant depression. J. Humanist. Psychol..

[80] Swift TC (2017). Cancer at the dinner table: Experiences of psychotherapy for the treatment of cancer-related distress. J. Humanist. Psychol..

[81] Gasser P, Kirchner K, Passie T (2015). LSD-assisted psychotherapy for anxiety associated with a life-threatening disease: A qualitative study of acute and sustained subjective effects. J. Psychopharmacol..

[82] Timmermann C (2018). DMT models the near-death experience. Front. Psychol..

[83] Turton S, Nutt DJ, Carhart-Harris RL (2014). A qualitative report on the subjective experience of intravenous psilocybin administered in an fMRI environment. Curr. Drug Abuse Rev..

[84] Varela FJ, Thompson E, Rosch E (1993). The embodied mind: Cognitive science and human experience.

[85] Studerus E, Gamma A, Vollenweider FX (2010). Psychometric evaluation of the Altered States of Consciousness rating scale (OAV). PLoS ONE.

[86] Wlodarczyk A (2020). Perceived emotional synchrony in collective gatherings: Validation of a short scale and proposition of an integrative measure. Front. Psychol..

[87] Tennant R (2007). The Warwick-Edinburgh mental well-being scale (WEMWBS): Development and UK validation. Health Qual. Life Outcomes.

[88] Carhart-Harris RL (2016). Psilocybin with psychological support for treatment-resistant depression: An open-label feasibility study. Lancet Psychiatry.

[89] Piedmont RL (2001). Spiritual transcendence and the scientific study of spirituality. J. Rehabil..

[90] Raîche G, Walls TA, Magis D, Riopel M, Blais JG (2013). Non-graphical solutions for Cattell’s scree test. Methodology.

[91] Kaiser HF (1960). The application of electronic computers to factor analysis. Educ. Psychol. Meas..

[92] Guadagnoli E, Velicer WF (1988). Relation of sample size to the stability of component patterns. Psychol. Bull..

