# Supplementary material for: Psychedelics alter metaphysical beliefs
Source: Sci Rep. 2021 Nov 23;11:22166. doi: 10.1038/s41598-021-01209-2 (PMC8611059; doi:10.1038/s41598-021-01209-2)
Supplement: Supplementary file 1 — Supplementary Information. [file 41598_2021_1209_MOESM1_ESM.pdf]

Supplementary Information for

Psychedelics alter metaphysical beliefs

Christopher Timmermann[1], Hannes Kettner[1], Chris Letheby[2, 3], Leor Roseman[1], Fernando E. Rosas[1,4,5] and Robin L. Carhart-Harris[1]

[1] Centre for Psychedelic Research, Division of Psychiatry, Department of Brain Sciences, Imperial College London
[2] Department of Philosophy, University of Western Australia
[3] Department of Philosophy, University of Adelaide
[4] Data Science Institute, Imperial College London
[5] Centre for Complexity Science, Imperial College London

Corresponding Author: Christopher Timmermann

E-mail address: c.timmermann-slater15@imperial.ac.uk

This PDF contains:

- Supplementary Methods
- Supplementary Results
- Supplementary Tables S1 to S2
- Supplementary Figures S1 to S4

Supplementary methods

Items used from scales

Items extracted from the Fatalistic Determinism subscale from the Free-Will and Determinism (FAD-plus) questionnaire[1] used for this study.

1. My future has already been determined by fate
2. No matter how hard you try, you can’t change your destiny
3. Fate already has a plan for each of us
4. What will be, will be-there’s not much you can do about it
5. Whether we like it or not, mysterious forces seem to move our lives

Items extracted from the Free-Will subscale from the FAD questionnaire[1] used for this study.

1. People have complete control over the decisions they make
2. People can overcome obstacles if they truly want to
3. Criminals are totally responsible for the bad things they do
4. Strength of mind can always overcome the body’s desires

Items comprising the ‘Universality’ subscale from the Spiritual Transcendence Scale[2] used for this study:

1. I feel that on a higher level all of us share a common bond.
2. All life is interconnected.
3. There is a higher plane of consciousness or spirituality that binds all people.

4. Although individual people may be difficult, I feel an emotional bond with all of humanity.
5. I believe that there is a larger meaning to life.
6. I believe that death is a doorway to another plane of existence.
7. I believe there is a larger plan to life.
8. There is an order to the universe that transcends human thinking.
9. I believe that on some level my life is intimately tied to all of humankind.

Supplementary results

Sample characteristics

At the time the data was extracted for analysis (February, 2020), 866 respondents had completed the measures at the baseline, 386 respondents completed the survey corresponding to 4 weeks post-retreat and 141 completed the questionnaires 6 months post-retreat. Supplementary Table 1 displays previous psychedelic use, the psychedelic used in the ceremony and the number of ceremonies attended for the planned retreat for a total of 866 respondents who completed the survey.

References

1. Paulhus, D. & Carey, J. The FAD-Plus : Measuring Lay Beliefs Regarding Free Will and Related Constructs The FAD – Plus : Measuring Lay Beliefs Regarding Free Will. *J. Pers. Assess.* **93**, 96–104 (2011).

2. Piedmont, R. L. Spiritual transcendence and the scientific study of spirituality. *J. Rehabil.* **67**, 4–14 (2001).

Supplementary Table 1. *Previous psychedelic use, psychedelic used in ceremony and number of ceremonies attended*

Total respondents		866
Previous psychedelic use	Never (psychedelic naïve)	330 (38.1%)
	Once	95 (11%)
	2-5 times	166 (19.2%)
	6-10 times	73 (8.4%)
	11-20 times	76 (8.8%)
	21-50 times	49 (5.7%)
	51-100 times	16 (1.8%)
	More than 100 times	14 (1.6%)
	No response	47 (5.4%)
Psychedelic used in ceremony	Psilocybin	656 (75.8%)
	Ayahuasca	128 (14.8%)
	Other	17 (2%)
	Combinations	10 (1%)
	No response	55 (6.4%)
Number of ceremonies per	1 ceremony	517 (59.5%)
retreat	2 ceremonies	88 (12%)
	3 ceremonies	78 (22.2%)
	More / No response	21 (3.7%)

Supplementary Table 2. *Recommended and actual fit indices for path modelling*

Fit index	Final model	Good fit	Acceptable fit
CFI	1.000	> .95	> .90
χ^2 /df	0.61	< 2.0	< 3.0
RMSEA	.000	< 0.05	< 0.08
SRMR	.016	< 0.05	< 0.08

Note. CFI: Comparative Fit Index, χ2: Chi-square test statistic, df: degrees of freedom, RMSEA: Root Mean Square Error of Approximation, SRMR: Standardized Root Mean Square Residual

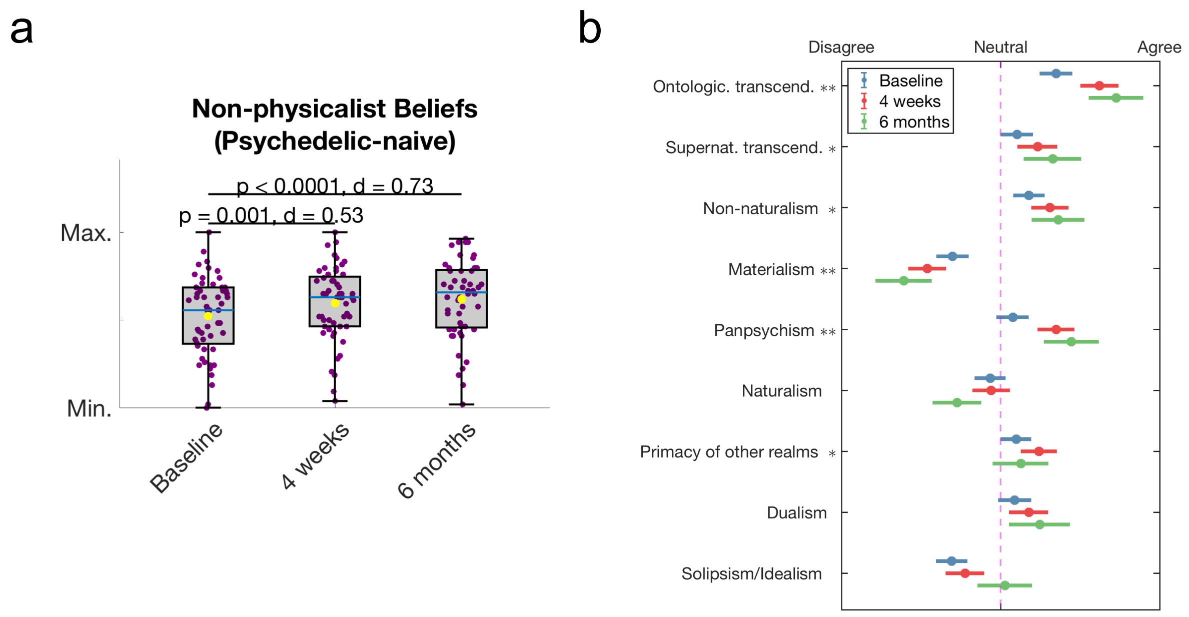

Supplementary Figure 1. Changes in Non-physicalist Beliefs for psychedelic-naïve respondents: (a) Significant increases were observed for Non-physicalist Beliefs which lasted up to 6 months for psychedelic-naïve respondents (Bonferroni-corrected values displayed). (b) Mean values and standard errors are displayed for each item comprising the Non-physicalist Beliefs factor (* = Significant change at 4 weeks; **= Significant change at 6 months, Bonferroni-corrected

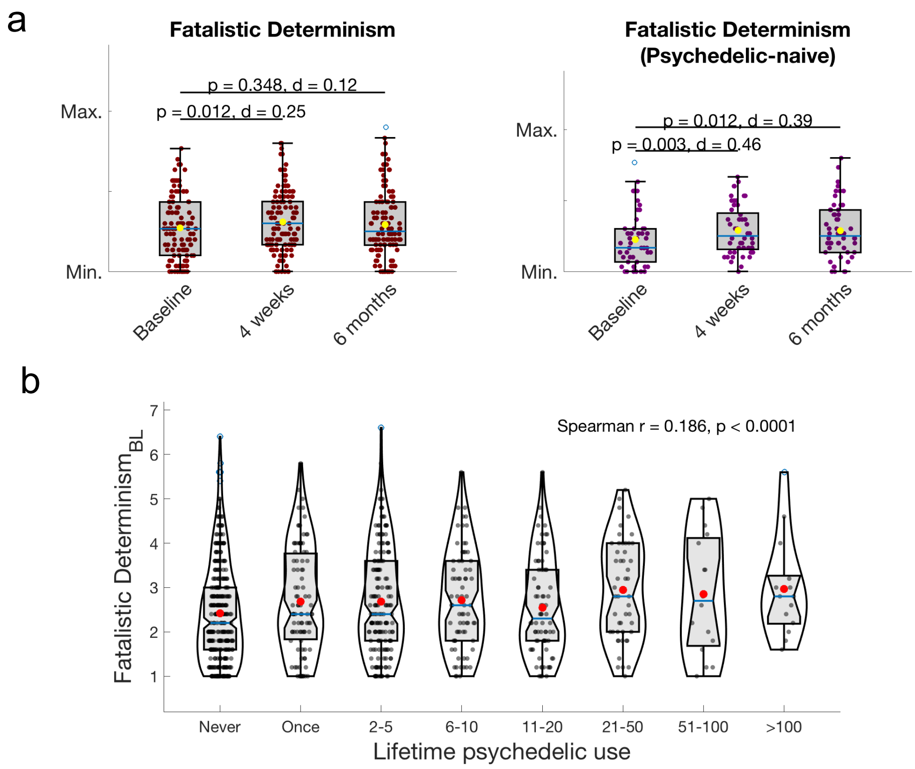

Supplementary Figure 2. Psychedelic use is associated with increases in beliefs associated with Fatalistic Determinism: Attending a psychedelic retreat was associated with increases in scores corresponding to endorsement of Fatalistic Determinism for 4 weeks post-retreat (a-left) and these changes endured for 6 months in respondents with no previous psychedelic experiences (a-right). A significant positive was observed between lifetime psychedelic use and baseline scores on Fatalistic Determinism (b). (Mean displayed in yellow/red and median in blue. Bonferroni-corrected for multiple comparisons)

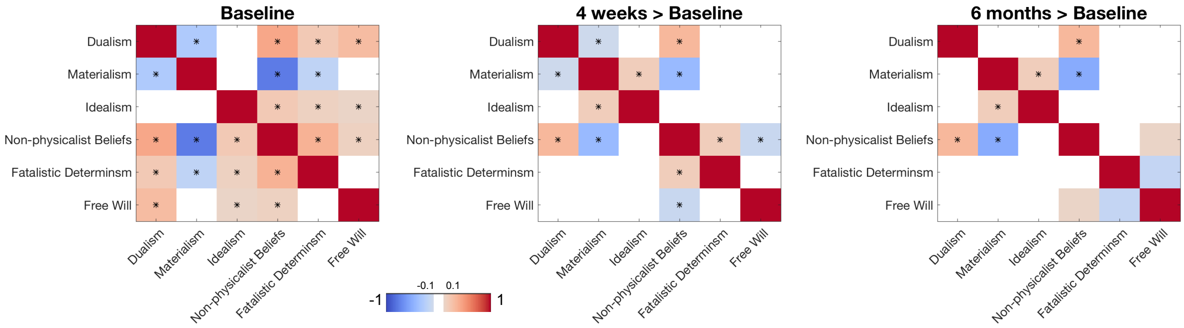

Supplementary Figure 3. Pearson correlation values between subscales and items of the Metaphysical Beliefs Questionnaire and the Fatalistic Determinism and Free Will subscales. (*p < 0.05, False-Discovery Rate-corrected for multiple comparisons. Items corresponding to Dualism and Materialism are omitted from the Non-physicalist Beliefs factor

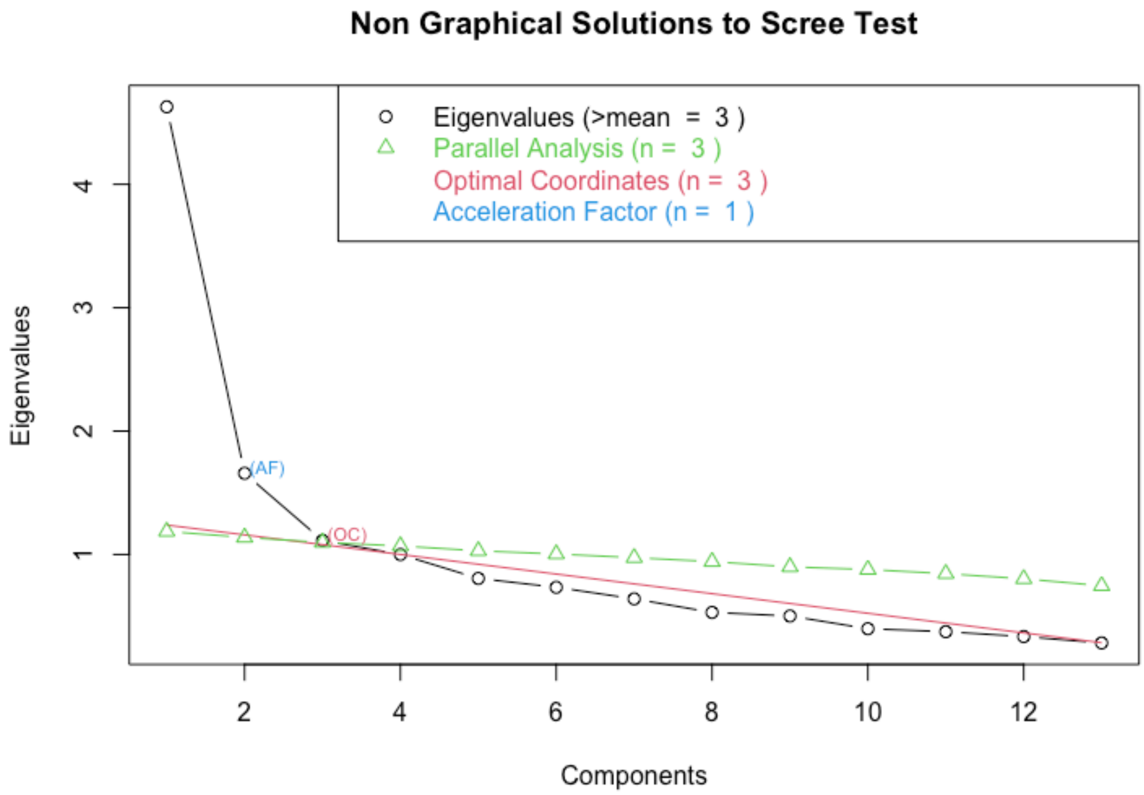

Supplementary Figure 4. Scree plot displaying eigenvalues and results of non-graphical tests to determine optimal number of factors to retain in the new Metaphysical Beliefs Questionnaire
